# Association of socioeconomic and lifestyle-related risk factors with mental health conditions: a cross-sectional study

**DOI:** 10.1186/s12889-019-8022-4

**Published:** 2019-12-30

**Authors:** Miwako Nagasu, Kazutaka Kogi, Isamu Yamamoto

**Affiliations:** 1 Faculty of Economics, Keio University, 〒108-8345 Tokyo-to, Minato-ku, Mita 2-15-45, Tokyo, Japan; 2 The Ohara Memorial Institute for Science of Labour, Tokyo, Japan; 3 Faculty of Business and Commerce, Keio University, Tokyo, Japan

**Keywords:** Mental health, Household disposable income, Socioeconomic status, Lifestyle, General Health questionnaire

## Abstract

**Background:**

There is rising public concern over the widening health inequalities in many countries. The aim of this study was to clarify the associations of socioeconomic status (SES)-related variables, such as levels of household disposable income and employment status, and lifestyle factors with mental health conditions among Japanese adults aged 40 to 69.

**Methods:**

A cross-sectional study of 3085 participants (1527 males and 1558 females) was undertaken by using a self-administered questionnaire that included the Japanese version of the 12-item General Health Questionnaire (GHQ-12) and questions related to socioeconomic and lifestyle factors.

**Results:**

The prevalence of poor mental health conditions, represented by a GHQ-12 score of 4 or more, was 33.4% among males and 40.4% among females. Males whose annual household disposable income was less than 2 million yen had significantly higher GHQ-12 scores than those with an annual household disposable income above 2 million yen. As per binary logistic regression analyses, short sleep duration and the absence of physical exercise were significantly related to poor mental health conditions among both males and females. Among females, a household disposable income of less than 2 million yen could be a risk factor for poor mental health conditions. Age and habitual drinking were inversely associated with poor mental health conditions.

**Conclusions:**

Low levels of household disposable income and unhealthy lifestyle factors were significantly associated with mental health conditions. These results suggest the importance of improving unhealthy lifestyle behaviours and developing effective health promotion programmes. In addition, there is a need for social security systems for people from different socioeconomic backgrounds.

## Background

The widening health inequalities associated with economic disparities have received significant attention in many countries, since previous studies have found that economic disparities associated with socioeconomic status (SES) may actually contribute to inequalities in health [1, 2]. Such studies have commonly taken account of income levels, employment status, occupations, and differences in educational levels, demonstrating such socioeconomic factors to be main determinants of both mental and physical health [3–5].

Mortality due to severe mental illnesses is a global public health concern [6]. In particular, severe mental illnesses can lead to suicide [7]. Kawakami et al. reported that the prevalence of mental disorders as assessed with the World Mental Health version of the World Health Organization (WHO) Composite International Diagnostic Interview (WMH-CIDI) and the Diagnostic and Statistical Manual of Mental Disorders 4th edition (DSM-IV) among the Japanese population was 8.8%, of which 17.0% of cases had severe mental disorders [8]. Moreover, Japan has one of the highest suicide rates in the world [9]. Qin et al. revealed that mental illnesses and SES-related variables, such as low income and unemployment, were significantly associated with higher suicide risk [10]. Two studies in the UK and Denmark also reported a strong association between current financial difficulties and mental disorders [11]. In addition, poor economic situation has been reported to be significantly associated with life dissatisfaction [12]. Therefore, the associations between mental health conditions, SES, and lifestyle factors have been receiving an increasing amount of attention [13].

The relationship between mental health and lifestyle factors such as sleep duration [14], habitual physical exercise [15, 16], smoking [17–19], and alcohol consumption has been studied. Among the risk factors for poor mental health, short sleep duration was reported to be linearly associated with psychological distress [14]. Low frequency of exercise [15, 16] and high smoking frequency [15] have also been known to significantly contribute to high scores on the 12-item General Health Questionnaire (GHQ-12). Regarding smoking habits, current and former smokers tended to experience more depressive symptoms than never smokers [17]. Taylor et al. reported that those who quit smoking displayed reduced depression, anxiety, and stress and improved mood and quality of life compared with current smokers [19]. Regarding alcohol consumption, it is a well-known cause of many major physical disease outcomes [20]. According to the WHO, alcohol consumption is responsible for approximately 5.9% of deaths worldwide [21]. Collins reported that people with lower SES seemed to bear a disproportionate burden of negative alcohol-related consequences [21]. In general, previous studies have reported linear relationships between mental health and each lifestyle factor. Therefore, this study adjusted for all potential risk factors in a single model to determine risk factors.

It has been reported that people with low SES are more likely to engage in unhealthy behaviours than those with high SES [20, 22]. For example, people with lower SES are more likely to be smokers, experiencing its negative impacts [22]. On the contrary, healthy lifestyle practices may be associated with a reduced risk of mental disorders. Lyu et al. reported that older South Koreans who engage in a greater number of healthy lifestyle practices have a higher overall level of health than those who do not engage in as many healthy lifestyle practices [23]. Identifying lifestyle-related risk factors would, thus, be beneficial in the promotion of improved mental health and might explain the background of income-related health inequalities.

As it has been consistently reported that the prevalence of mental illnesses and lifestyle-related factors differs between males and females, in the present study, data analysis was stratified by gender [24]. Matud et al. reported that adherence to traditional gender roles had a great impact on psychological well-being [22]. With regard to lifestyle, one specific factor—sleep duration—is a risk factor for depression that has an inherent gender difference; it has been reported that females sleep longer than males. In particular, being pregnant has been associated with longer sleep hours [24]. The gender differences in healthy lifestyles could potentially play confounding roles in the determination of the association between mental health and lifestyle factors, necessitating gender stratification of analyses.

In this study, we analysed a large nationally representative data set and hypothesised that SES and lifestyle-related factors would be significantly associated with mental health conditions, even after controlling for all potential risk factors. Moreover, the associations for both genders were examined separately. Therefore, the objectives of this study were to (1) investigate the prevalence of psychological distress (GHQ-12 score ≥ 4) among Japanese people aged 40 to 69 and (2) separately identify the associations of SES and lifestyle-related factors with poor mental health conditions among males and females.

## Methods

This study used the data set in 2017 from the Japan Household Panel Survey (JHPS/KHPS) as a cross-sectional data set. This JHPS/KHPS date set has combined two longitudinal panel data set: the JHPS (the Japan Household Panel Survey) and the KHPS (the Keio Household Panel Survey). The data set used, the JHPS/KHPS in 2017, was collected from February to March 2017. The key characteristics of panel studies is that they collect repeated measures from the same participants at different points. The questionnaire for the study in 2017 was distributed to 5030 respondents who participated in the JHPS/KHPS survey in 2016. Of these 5030 respondents, 4626 (92.0%) returned completed questionnaires. In the final analysis of this study, 1541 questionnaires were excluded either because the respondents did not fall within the 40–69 age range or because of incomplete information regarding gender, age, or other important variables. Finally, the data of 3085 respondents were used for analysis (valid response rate: 61.3%). An informed consent form consisting of the aims and details of the study, as well as an assurance regarding anonymity and confidentiality, was sent to all respondents. Those who provided informed consent received the questionnaire.

### Sampling

The JHPS/KHPS respondents, selected through two-stage stratified random sampling. According to the National Census Survey, Japan is divided into 24 strata, from which 354 research areas were randomly selected. Five to 10 individuals from each research area were selected by using population ratios based on basic resident register information. The KHPS began with 4005 respondents in 2004. In 2007 and 2012, 1400 and 1000 respondents, respectively, were added. As for the JHPS, it began with 4022 respondents in 2009. From 2004 to 2017, a total of 10,458 respondents participated. Over the course of 13 years, the number of respondents declined gradually, and new respondents were added to ensure the survey remained representative of Japan’s population distribution.

### Study variables

A self-administered questionnaire was used to collect information about socioeconomic factors, lifestyle-related factors, and mental health outcomes.

#### Socioeconomic factors

The socioeconomic factors included gender, age, employment status, number of persons in the household, and the level of disposable income per household and respondent. Based on age, the participants were divided into three groups: 40–49, 50–59, and 60–69. On the basis of employment status, two groups were created: full-time workers and others (self-employed persons, freelance professionals, part-time workers, and unemployed). The number of persons in the household was categorized as two or more (living with someone) and one (living alone). All respondents provided information regarding disposable income—both the family’s and their own—in the last year. This disposable income excluded tax and social insurance fees. The level of disposable income per household was divided into three groups: less than two million yen, two million yen to under six million yen, and six million yen and above. Respondents’ level of disposable income was divided into three groups: less than two million yen, two million yen to under five million yen, and five million yen and above.

#### Health outcomes

The Japanese version of the GHQ-12, used as a screening tool to detect nonpsychotic psychiatric diseases, consists of 12 questions about feelings over the past few weeks. The questions include the following: Have you recently (1) been able to concentrate on whatever you’re doing, (2) lost much sleep over worry, (3) felt that you were playing a useful part in things, (4) felt capable of making decisions, (5) felt constantly under strain, (6) felt you couldn’t overcome your difficulties, (7) been able to enjoy your normal day-to-day activities, (8) been able to face problems, (9) been feeling unhappy or depressed, (10) been losing confidence in yourself, (11) been thinking of yourself as a worthless person, and (12) been feeling reasonably happy, all things considered. The scoring system used was as follows: the response categories (1, 2, 3, and 4) were converted into corresponding binary values (0, 0, 1, and 1) to calculate the total score. The sum of the scores indicated the severity of psychological distress. The participants were then divided into two groups: those with high scores (poor mental health conditions: ≥ 4 points) and those with low scores (good mental health conditions: ≤ 3 points) [25]. Goldberg reported that sensitivity and specificity ranged from 67.0 to 93.5% (median 83.7%) and 59.0 to 93.0% (median 79.0%), respectively, when the cut-off point is between under 3 and 4 points and more as a screening tool of psychological distress [26].

#### Lifestyle factors

This study included five lifestyle-related questions for assessing lifestyle practices: sleep duration on weekdays, physical exercise frequency, and smoking and drinking habits. About sleep duration on weekdays, the question was “How many hours do you usually sleep on weekdays?” The responses were categorised into three groups: 7 h or more, 6–7 h, and fewer than 6 h. Physical exercise frequency was determined by the question: “How many days per week do you engage in physical exercise that makes you sweat (excluding your work)?” The answers were categorised into three groups: three or more days, two or fewer days, and no exercise. By smoking habit, respondents were categorised as never smokers, ex-smokers, or current smokers. Alcohol consumption was assessed by the question: “How often do you drink alcohol?” The answers were categorised into the following groups: never, two or fewer times/week, and three or more times/week.

### Statistical methods

The gender-stratified association of socioeconomic and lifestyle factors with mental health conditions was analysed by Pearson’s chi-squared test. The differences in GHQ-12 scores in household disposable income groups stratified by gender were analysed by the Kruskal-Wallis test with the Bonferroni correction and the Student’s *t*-test. *P* values less than 0.05 indicated statistical significance. We investigated the adjusted prevalence odds ratios (AORs) and 95% confidence intervals (CI) of high GHQ-12 scores (≥ 4 points) by using binary logistic regression analysis. All factors such as age, number of persons in the household, employment status, annual disposable income per household, and lifestyle-related factors were adjusted for. The data were analysed by gender, annual disposable income per household, and employment status. SPSS 24.0 was used for data analysis.

## Results

This study included 3085 respondents, of which 1527 (49.5%) were males and 1558 (50.5%) were females. The mean age of the males (54.9 ± 8.8 years) was similar to that of the females (54.5 ± 8.7 years). Table 1 depicts the gender-specific distribution of socioeconomic, lifestyle, and mental health variables. The results indicated statistically significant gender differences in the ratios of the corresponding categories of all variables except age and disposable household income. The rate of those living alone was 10.5% among males and 7.3% among females. Regarding employment status, 62.8% of the males worked full time, while this figure stood at 21.8% among females. In the context of socioeconomic variables, of those with an annual disposable income of less than two million yen per respondent, 13.6% were males and 66.2% were females. As for lifestyle-related variables, more females than males had short sleep durations (< 6 h), no exercise, and no smoking and alcohol consumption; these differences were statistically significant. The overall rate of those with a high GHQ-12 score was 36.9%. Stratified by gender, 33.4% of the males and 40.4% of the females, respectively, had high GHQ-12 scores. These gender differences were statistically significant.

**Table 1:** Demographic characteristics of the respondents by gender

		Total		Males		Females		*P* value^1)^
Variables	Group	n	%	n	%	n	%	
Gender	Males	1527	49.5					
	Females	1558	50.5					
Age (years)	40–49	1037	33.6	504	33.0	533	34.2	
	50–59	1018	33.0	490	32.1	528	33.9	n.s
	60–69	1030	33.4	533	34.9	497	31.9	
Number of persons in the household	≥2	2742	91.2	1325	89.5	1417	92.7	
	1 person	266	8.8	155	10.5	111	7.3	^**^
Employment status	Full time	1083	44.5	845	62.8	238	21.8	
	Others	1353	55.5	501	37.2	852	78.2	^***^
Disposable income per household (JPY)	< 2000 K	287	9.3	129	8.4	158	10.1	
	2000 K–< 6000 K	1479	47.9	746	48.9	733	47.0	n.s.
	≥ 6000 K	1319	42.8	652	42.7	667	42.8	
Disposable income of the respondent (JPY)	< 2000 K	880	37.1	178	13.6	702	66.2	
	2000 K–< 5000 K	805	33.9	515	39.2	290	27.3	^***^
	≥ 5000 K	689	29.0	620	47.2	69	6.5	
Sleep duration on weekdays (hours)	≥ 7	1241	40.4	656	43.2	585	37.7	
	6–7	1166	38.0	563	37.1	603	38.9	^**^
	< 6	663	21.6	299	19.7	364	23.5	
Physical exercise (days/week)	≥ 3	429	14.0	225	14.9	204	13.2	
	≤ 2	530	17.3	293	19.4	237	15.3	^**^
	No exercise	2097	68.6	994	65.7	1103	71.4	
Smoking	Never	1605	52.2	449	29.5	1156	74.5	
	Quit	836	27.2	610	40.1	226	14.6	^***^
	Sometimes/everyday	633	20.6	463	30.4	170	11.0	
Drinking (times/week)	Never	1168	38.0	391	25.7	777	50.0	
	≤ 2	898	29.2	401	26.4	497	32.0	^***^
	≥ 3	1008	32.8	729	47.9	279	18.0	
GHQ-12 score	≥ 4 (poor)	1131	36.9	506	33.4	625	40.4	^***^
	≤ 3	1933	63.1	1010	66.6	923	59.6	

^1)^ *P* value from Pearson’s chi-squared test: ** *P* < 0.01, *** *P* < 0.001

Table 2 depicts the differences in GHQ-12 scores by gender; in particular, in terms of stratified disposable income per household and employment status. Male respondents with a household disposable income of less than two million yen had significantly higher GHQ-12 scores than those in the higher-income groups. This tendency was not statistically significant among the females.

**Table 2:** Differences in GHQ-12 scores by gender, disposable income per household, and employment status

Disposable income per household						*P* value^1)^		Employment status				*P* value^2)^
< 2000 K		2000 K – < 6000 K		≥ 6000 K		2000 K – < 6000 K	≥ 6000 K	Full-time		Others		
mean	SD	mean	SD	mean	SD			mean	SD	mean	SD	
Males												
3.992	4.007	2.878	3.295	2.981	3.402	^*^	^*^	3.033	3.360	2.879	3.405	n.s.
Females												
3.790	3.351	3.480	3.340	3.325	3.410	n.s.	n.s.	3.705	3.372	3.579	3.355	n.s.

^1)^ The Shapiro-Wilk test was used to assess normality of data distribution for GHQ-12 scores. Comparing GHQ-12 scores between three groups (Ref: Household disposable income < 2000 K). *P* value from Kruskal-Wallis test with Bonferroni correction: ^*^P < 0.05

^2)^ Mann–Whitney U test

As Table 3 depicts, household disposable income levels and employment status were associated with mental health conditions and lifestyle factors. Among all respondents, though it was particularly true of males, earning less than two million yen was associated with living alone and not having a full-time job. Regarding lifestyle factors, males with a household disposable income over six million yen had shorter sleep durations than those who earned less than six million yen. About habitual physical exercise, males with a household disposable income of six million yen and above had a higher exercise frequency (≥ 3 days/week) than those with a household disposable income under two million yen. Male respondents with a household disposable income under two million yen were less likely to engage in physical exercise than those in higher-income categories. About smoking habits, both males and females who were categorised into the group with a household disposable income under two million yen were significantly more likely to be smokers than those categorised into the group with a household disposable income of six million yen and above. About alcohol consumption, male respondents with a household disposable income under two million yen had a significantly lower alcohol consumption frequency (≥ 3 times/week) than those with a household disposable income of 6 million yen and above.

**Table 3:** Associations between lifestyle and socioeconomic factors by gender

Variables	Men												Women											
	Disposable income per household							Employment status					Disposable income per household							Employment status				
	< 2000 K		2000 K – < 6000 K		≥ 6000 K			Full time		Others			< 2000 K		2000 K – < 6000 K		≥ 6000 K			Full time		Others		
	n	%	n	%	n	%	*P* value^1)^	n	%	n	%	*P* value^1)^	n	%	n	%	n	%	*P* value^1)^	n	%	n	%	*P* value^1)^
Age (years)																								
40–49	26	20.2	254	34.0	224	34.4		383	45.3	102	20.4		44	27.8	246	33.6	243	36.4		105	44.1	309	36.3	
50–59	32	24.8	186	24.9	272	41.7	^***^	354	41.9	120	24.0		44	27.8	237	32.3	247	37.0	^***^	103	43.3	314	36.9	^***^
60–69	71	55.0	306	41.0	156	23.9		108	12.8	279	55.7	^***^	70	44.3	250	34.1	177	26.5		30	12.6	229	26.9	
Number of persons in the household																								
≥ 2	73	57.5	649	90.0	603	95.4		749	91.7	432	88.3		107	67.7	682	94.7	628	96.6		214	91.8	777	93.1	n.s.
1	54	42.5	72	10.0	29	4.6	^***^	68	8.3	57	11.7	^*^	51	32.3	38	5.3	22	3.4	^***^	19	8.2	58	6.9	
Employment status																								
Full time	30	35.7	371	56.2	444	73.8							17	15.0	89	17.8	132	27.7						
Others	54	64.3	289	43.8	158	26.2	^***^						96	85.0	412	82.2	344	72.3	^***^					
Sleep duration on weekdays (hours)																								
≥ 7	70	54.3	335	45.1	251	38.8		275	32.8	269	53.8		67	42.7	302	41.3	216	32.6		68	28.8	299	35.3	
6–7	43	33.3	270	36.4	250	38.6	^**^	355	42.3	157	31.4	^***^	59	37.6	281	38.4	263	39.7	^**^	93	39.4	344	40.6	^*^
< 6	16	12.4	137	18.5	146	22.6		209	24.9	74	14.8		31	19.7	149	20.4	184	27.8		75	31.8	205	24.2	
Physical exercise (days/week)																								
≥ 3	16	12.4	104	14.1	105	16.3		91	10.9	85	17.1		24	15.4	100	13.7	80	12.1		13	5.5	94	11.1	
≤ 2	19	14.7	109	14.7	165	25.6	^***^	191	22.8	85	17.1	^**^	22	14.1	101	13.9	114	17.3	n.s.	36	15.3	143	16.9	^*^
No exercise	94	72.9	526	71.2	374	58.1		555	66.3	328	65.9		110	70.5	528	72.4	465	70.6		186	79.1	611	72.1	
Smoking																								
Never	28	21.7	202	27.1	219	33.8		268	31.9	129	25.7		99	63.1	544	74.5	513	77.1		152	65.0	622	73.1	
Quit	49	38.0	307	41.2	254	39.2	^**^	304	36.1	217	43.3	^*^	29	18.5	111	15.2	86	12.9	^**^	43	18.4	141	16.6	^*^
Sometimes/everyday	52	40.3	236	31.7	175	27.0		269	32.0	155	30.9		29	18.5	75	10.3	66	9.9		39	16.7	88	10.3	
Drinking (times/week)																								
0	49	38.0	203	27.2	139	21.5		197	23.5	118	23.6		91	57.6	371	50.7	315	47.5		108	45.8	404	47.6	
≤ 2	27	20.9	202	27.1	172	26.6	^**^	251	29.9	116	23.2	^*^	37	23.4	236	32.2	224	33.8	n.s.	87	36.9	279	32.9	n.s.
≥ 3	53	41.1	340	45.6	336	51.9		392	46.7	267	53.3		30	19.0	125	17.1	124	18.7		41	17.4	166	19.6	
GHQ-12 score																								
≥ 4 (poor)	55	43.0	245	33.0	206	31.9	n.s	284	33.8	156	31.4	n.s.	71	45.2	296	40.5	258	39.0	n.s.	128	54.7	360	42.4	n.s.
≤ 3	73	57.0	497	67.0	440	68.1		556	66.2	341	68.6		86	54.8	434	59.5	403	61.0		106	45.3	490	57.6	

^1)^ *P* value from Pearson’s chi-squared test: ^*^P < .05, ^**^ *P* < 0.01, ^***^ *P* < 0.001

Tables 4 and 5 depict the rates of those with high GHQ-12 scores (≥ 4 points), crude odds ratios (OR) and adjusted odds ratios (AOR) by using binary logistic regression analyses. Males who lived alone and had a disposable income of six million yen or above showed significantly higher AORs (AOR 2.548 [95% CI: 1.049–6.189]). About lifestyle-related factors, short sleep duration (< 6 h) had a significantly greater association with poor mental health than sleeping over 6 h per day (males in total: AOR 1.613 [95% CI: 1.171–2.221], males with income 2 million–≥6 million: AOR 2.199 [95% CI: 1.383–3.498], males without full-time work: AOR 1.837 [95% CI: 0.531–6.363]). Lack of physical exercise was also significantly associated with poor mental health conditions (males in total: AOR 1.663 [95% CI: 1.122–2.465], males with income ≥6 million: 1.743 [95% CI: 0.996–3.048], males without full-time work: AOR 5.175 [95% CI: 2.014–13.295]). Therefore, sleeping for fewer than 6 h and a lack of physical exercise could be risk factors for poor mental health among males.

**Table 4:** Prevalence and associations of the GHQ-12 score with risk factors among males

	Males								Disposable income per household									Employment status					
									< 2000 K			2000 K – < 6000 K			≥ 6000 K			Full time			Others		
	Prevalence (≥ 4)^a)^		Crude OR ^b)^	95% CI		Adjusted OR^c)^	95% CI		Adjusted OR^d)^	95% CI		Adjusted OR^d)^	95% CI		Adjusted OR^d)^	95% CI		Adjusted OR^e)^	95% CI		Adjusted OR^e)^	95% CI	
	n	%		Lower	Upper		Lower	Upper		Lower	Upper		Lower	Upper		Lower	Upper		Lower	Upper		Lower	Upper
Age (years)																							
40–49	167	33.3	1			1			1			1			1			1			1		
50–59	177	36.3	1.145	0.881	1.488	1.153	0.870	1.528	0.872	0.249	3.049	1.344	0.879	2.055	1.096	0.729	1.647	1.258	0.908	1.742	1.267	0.269	5.970
60–69	165	30.7	0.890	0.685	1.157	0.908	0.650	1.268	1.101	0.339	3.576	0.781	0.482	1.266	1.191	0.699	2.026	0.845	0.515	1.387	0.391	0.125	1.221
Number of persons in the household																							
≥ 2	427	32.4	1			1			1			1			1			1			1		
1	68	45.0	**1.711**	**1.217**	**2.406**	1.495	0.992	2.253	1.237	0.465	3.286	1.220	0.702	2.123	**2.548**	**1.049**	**6.189**	1.418	0.830	2.421	1.498	0.566	3.962
Employment status																							
Full time	284	33.8	1			1			1			1			1			NA^f)^			NA^f)^		
Others	156	31.4	0.896	0.706	1.136	0.970	0.732	1.284	0.993	0.380	2.593	0.937	0.626	1.402	1.002	0.640	1.568	NA^f)^			NA^f)^		
Household disposable income (JPY)																							
≥ 6000 K	206	31.9	1			1			NA^f)^			NA^f)^			NA^f)^			1			1		
2000 K–< 6000 K	245	33.0	1.053	0.840	1.319	1.027	0.793	1.331	NA^f)^			NA^f)^			NA^f)^			1.177	0.852	1.626	0.682	0.291	1.594
< 2000 K	55	43.0	**1.609**	**1.093**	**2.370**	1.535	0.918	2.567	NA^f)^			NA^f)^			NA^f)^			1.693	0.770	3.723	0.714	0.263	1.939
Sleep duration on weekdays (hours)																							
≥ 7	208	31.9	1			1			1			1			1			1			1		
6–7	171	30.5	0.938	0.735	1.197	0.925	0.700	1.221	1.080	0.367	3.181	1.110	0.744	1.655	0.735	0.478	1.132	1.035	0.723	1.481	1.019	0.471	2.203
< 6	126	42.3	**1.564**	**1.179**	**2.075**	**1.613**	**1.171**	**2.221**	0.985	0.253	3.830	**2.199**	**1.383**	**3.498**	1.233	0.762	1.995	1.460	0.976	2.182	**1.837**	**0.531**	**6.363**
Physical exercise (days/week)																							
≥ 3	49	21.9	1			1			1			1			1			1			1		
≤ 2	86	29.5	1.491	0.995	2.234	1.250	0.795	1.964	1.061	0.147	7.680	1.101	0.530	2.288	1.352	0.728	2.511	1.030	0.575	1.846	7.551	1.885	30.247
0	367	37.1	**2.111**	**1.499**	**2.971**	**1.663**	**1.122**	**2.465**	1.037	0.182	5.925	1.588	0.870	2.897	**1.743**	**0.996**	**3.048**	1.497	0.885	2.533	**5.175**	**2.014**	**13.295**
Smoking																							
Never	153	34.1	1			1			1			1			1			1			1		
Quit	197	32.6	0.936	0.723	1.213	0.929	0.693	1.246	0.984	0.257	3.770	0.848	0.549	1.311	1.012	0.660	1.553	1.030	0.715	1.485	1.315	0.587	2.945
Sometimes/everyday	155	33.6	0.980	0.745	1.290	0.927	0.682	1.261	1.507	0.443	5.128	0.784	0.494	1.244	1.010	0.638	1.597	0.926	0.631	1.357	1.549	0.583	4.112
Drinking (times/week)																							
0	141	36.2	1			1			1			1			1			1			1		
≤ 2	122	30.5	0.772	0.574	1.039	0.881	0.632	1.228	1.306	0.364	4.686	0.910	0.573	1.445	0.745	0.437	1.268	0.910	0.602	1.376	0.714	0.261	1.952
≥ 3	241	33.3	0.878	0.678	1.136	1.055	0.780	1.426	1.171	0.369	3.710	1.081	0.701	1.668	1.013	0.631	1.626	1.136	0.771	1.673	0.878	0.404	1.907

^a^) Prevalence of GHQ-12 score ≥ 4

^b^) Crude odds ratio with 95% confidence interval (no adjustment for any variables)

^c^) Adjusted odds ratios with 95% confidence interval (adjusted for age, number of persons in the household, employment status, disposable income per household, sleep duration on weekdays, physical exercise, smoking, and drinking)

^d^) Adjusted odds ratios with 95% confidence interval (adjusted for age, number of persons in the household, employment status, sleep duration on weekdays, physical exercise, smoking, and drinking)

^e^) Adjusted odds ratios with 95% confidence interval (adjusted for age, number of persons in the household, disposable income per household, sleep duration on weekdays, physical exercise, smoking, and drinking)

^f^) *NA* not applicable

^g^) Bold ratios: statistically siginificant results

**Table 5:** Prevalence and associations of the GHQ-12 score with risk factors among females

	Females								Disposable income per household									Employment status					
									< 2000 K			2000 K – < 6000 K			≥ 6000 K			Full time			Others		
	Prevalence (≥ 4)^a)^		Crude OR^b)^	95% CI		Adjusted OR ^c)^	95% CI		Adjusted OR ^d)^	95% CI		Adjusted OR ^d)^	95% CI		Adjusted OR ^d)^	95% CI		Adjusted OR^e)^	95% CI		Adjusted OR ^e)^	95% CI	
	n	%		Lower	Upper		Lower	Upper		Lower	Upper		Lower	Upper		Lower	Upper		Lower	Upper		Lower	Upper
Age (years)																							
40–49	258	48.5	1			1						1			1			1			1		
50–59	204	38.9	**0.675**	**0.529**	**0.862**	**0.680**	**0.511**	**0.904**	0.830	0.296	2.333	0.682	0.442	1.053	**0.641**	**0.422**	**0.974**	0.635	0.352	1.147	**0.577**	**0.328**	**1.017**
60–69	163	33.2	**0.528**	**0.410**	**0.680**	**0.676**	**0.479**	**0.954**	0.606	0.218	1.685	0.823	0.503	1.345	**0.512**	**0.283**	**0.926**	**0.257**	**0.085**	**0.781**	0.521	0.315	0.862
Number of persons in the household																							
≥ 2	585	41.6	1			1			1			1			1			1			1		
1	33	29.7	**0.594**	**0.390**	**0.905**	0.643	0.371	1.113	0.507	0.201	1.279	0.485	0.201	1.171	1.186	0.321	4.384	1.021	0.311	3.351	0.478	0.187	1.221
Employment status																							
Full time	106	45.3	1			1			1			1			1			NA^f)^			NA^f)^		
Others	360	42.4	0.887	0.663	1.187	0.970	0.709	1.327	1.865	0.559	6.219	0.697	0.422	1.153	1.164	0.747	1.815	NA^f)^			NA^f)^		
Household disposable income (JPY)																							
≥ 6000 K	258	39.0	1			1			NA^f)^			NA^f)^			NA^f)^			1			1		
2000 K–< 6000 K	296	40.4	1.065	0.859	1.321	1.128	0.862	1.477	NA^f)^			NA^f)^			NA^f)^			1.624	0.499	5.293	0.579	0.277	1.209
< 2000 K	71	45.2	1.290	0.908	1.832	**1.592**	**1.000**	**2.535**	NA^f)^			NA^f)^			NA^f)^			0.923	0.287	2.966	**0.567**	**0.268**	**1.203**
Sleep duration on weekdays (hours)																							
≥ 7	200	34.4	1			1			1			1			1			1			1		
6–7	247	41.2	**1.337**	**1.056**	**1.693**	1.238	0.920	1.665	1.252	0.469	3.341	1.166	0.758	1.795	1.632	0.735	3.624	1.546	0.767	3.119	1.357	0.860	2.141
< 6	176	48.6	**1.803**	**1.379**	**2.356**	**1.505**	**1.077**	**2.104**	1.158	0.377	3.563	1.568	0.954	2.575	1.767	0.877	3.561	**2.288**	**1.103**	**4.746**	2.632	1.492	4.643
Physical exercise (days/week)																							
≥ 3	60	29.6	1			1			1			1			1			1			1		
≤ 2	83	35.0	1.285	0.859	1.921	1.150	0.681	1.941	1.236	0.240	6.353	0.698	0.311	1.570	1.635	0.736	3.631	2.763	0.585	13.055	1.559	0.738	3.296
0	479	43.5	**1.838**	**1.329**	**2.542**	**1.738**	**1.120**	**2.696**	1.171	0.318	4.308	1.804	0.947	3.433	1.755	0.871	3.537	2.364	0.579	9.648	**1.298**	**0.754**	**2.235**
Smoking																							
Never	457	39.7	1			1			1			1			1			1			1		
Quit	97	43.3	1.158	0.867	1.547	0.979	0.696	1.376	1.028	0.341	3.098	0.894	0.542	1.475	1.180	0.694	2.006	1.240	0.591	2.602	1.682	0.834	3.391
Sometimes/everyday	68	40.5	1.031	0.741	1.434	0.999	0.668	1.495	0.934	0.275	3.170	1.163	0.642	2.108	0.864	0.448	1.666	0.978	0.440	2.173	0.712	0.340	1.489
Drinking (times/week)																							
0	328	42.5	1			1			1			1			1			1			1		
≤ 2	190	38.4	0.841	0.668	1.060	0.861	0.648	1.143	**0.274**	**0.100**	**0.751**	0.939	0.615	1.433	1.085	0.704	1.671	0.996	0.532	1.864	0.621	0.378	1.022
≥ 3	105	37.8	0.820	0.619	1.086	0.738	0.518	1.051	**0.289**	**0.092**	**0.906**	0.727	0.431	1.225	0.888	0.511	1.543	0.651	0.282	1.501	1.286	0.721	2.293

^a^) Prevalence of GHQ-12 score ≥ 4

^b^) Crude odds ratio with 95% confidence interval (no adjustment for any variables)

^c^) Adjusted odds ratios with 95% confidence interval (adjusted for age, number of persons in the household, employment status, disposable income per household, sleep duration on weekdays, physical exercise, smoking, and drinking)

^d^) Adjusted odds ratios with 95% confidence interval (adjusted for age, number of persons in the household, employment status, sleep duration on weekdays, physical exercise, smoking, and drinking)

^e^) Adjusted odds ratios with 95% confidence interval (adjusted for age, number of persons in the household, disposable income per household, sleep duration on weekdays, physical exercise, smoking, and drinking)

^f^) *NA* not applicable

^g^) Bold ratios: statistically siginificant

Among females, household disposable income (< 2 million yen: AOR 1.592 [95% CI: 1.000–2.535]) was significantly associated with poor mental health conditions. About lifestyle factors, female respondents with short sleep duration (< 6 h) (females in total: AOR 1.505 [95% CI: 1.077–2.104], full-time work: AOR 2.288 [95% CI: 1.103–4.746]) and without physical exercise (females in total: AOR 1.738 [95% CI: 1.120–2.696], non-full-time work: AOR 1.298 [95% CI: 0.754–2.235]) also had significantly higher rates of those with poor mental health conditions as compared with respondents who slept over 6 h per day and engaged in physical exercise.

However, the results show the existence of an inverse association between age and alcohol consumption and poor mental health. The following AOR levels were statistically significant (females overall aged 50–59: AOR 0.680 [95% CI: 0.511–0.904], aged 60–69: AOR 0.676 [95% CI: 0.479–0.954], females with ≥6 million aged 50–59: AOR 0.641 [95% CI: 0.422–0.974], aged 60–69: AOR 0.512 [95% CI: 0.283–0.926], working full-time aged 60–69: AOR 0.257 [95% CI: 0.085–0.781], not working full-time aged 50–59: AOR 0.577 [95% CI: 0.328–1.017]). Concerning alcohol consumption, among females with an income under two million yen, there was an inverse association between frequent drinking and mental health conditions (≤ 2 times/week AOR 0.274 [95% CI: 0.100–0.751], ≥ 3 times/week: AOR 0.289 [95% CI: 0.092–0.906]).

## Discussion

The results of this study revealed that males with low household disposable income (< 2 million yen) showed psychological distress with the highest GHQ-12 scores among three income levels. Moreover, according to the results of binary logistic regression analyses after controlling for all covariates, the potential risk factors among males and females differed by household disposable income levels and employment status. Low income was a significant risk factor for psychological distress in females. About lifestyle factors, short sleep duration on weekdays and a lack of physical exercise were significant risk factors for psychological distress in both males and females. Among males at the middle level of household disposable income, there was an association between short sleep duration and psychological distress. Among males with a high level of household disposable income, the number of persons in the household and a lack of physical exercise were associated with psychological distress. Among males who did not have full-time jobs, short sleep duration and a lack of physical exercise were potential risk factors for psychological distress. Regarding females, low levels of household disposable income, short sleep duration, and no physical exercise were risk factors for psychological distress. Short sleep duration and a lack of physical exercise were risk factors for full-time female workers and non-full time female workers, respectively. According to these results, potential risk factors for psychological distress differ by gender, disposable income levels, and employment status.

Comparing GHQ-12 scores between the three disposable income levels, males with a disposable income of less than two million yen had the lowest scores (3.992 ± 4.007). Hori et al. reported GHQ-12 scores of 2.34 ± 3.14 in the general male population aged 40–64 [27]. In this study, low-income males had higher GHQ-12 scores than the general population.

Moreover, after controlling for all covariates, females with low income levels had a significant association with poor mental health conditions. In previous studies, lower income group had the highest GHQ scores because financial hardship could be a cause of poor mental health conditions [28, 29]. Two cohort studies in the UK and Denmark also reported a strong association between current economic difficulties and mental disorders [11]. Therefore, both cross-sectional and longitudinal studies have demonstrated a significant association between poor economic situation and psychological disorders. Accordingly, addressing the mechanism linking socioeconomic factors with mental health outcomes is a necessity. In particular, enhancing social security systems and organising effective health promotion programmes would be helpful for people from different socioeconomic backgrounds.

After controlling for all variables, lifestyle factors such as shorter sleep duration and lack of physical exercise were significant potential risk factors for poor mental health conditions. Previous studies on adults have also suggested the significant association of short sleep duration [24, 27, 30] with depression [24], anxiety [31, 32], and poorer GHQ-12 scores [33], as well as the fact that it is a risk factor for later depression [34]. Thus, short sleep duration could be a cause of poor mental health conditions [31]. Our findings suggest that sleeping for more than 7 h per day is critical for preventing psychological distress.

Regarding habitual physical exercise, males with high incomes showed a higher tendency than those with low income. Among both males and females, especially males with a household disposable income of six million yen and above and non-full-time workers, the lack of physical activity could be a potential risk factor for psychological distress. Some studies have reported that physical activity is associated with better mental health outcomes [16, 27, 35]. There is, thus, a need to promote engagement in physical activity to reduce stress and improve mental health.

About alcohol consumption, while both males and females with lower income tended to drink less alcohol than those with higher income, this was especially true of male respondents. This result was consistent with previous findings [36, 37]. The Japanese National Survey reported that male respondents with lower income tended to drink less alcohol than those with high income [36]. The Centers for Disease Control and Prevention also reported that the prevalence of heavy episodic drinking increased with increase in household income [37]. However, people with lower SES tended to experience more negative alcohol-related outcomes than people with higher SES [38]. This implies that while people with lower SES may be less likely to drink alcohol, their health conditions are more negatively affected by unhealthy alcohol drinking habits. More research is necessary to address the long-term associations between drinking habits and health outcomes depending on SES.

As depicted in Table 5, it is noteworthy that one out of two female respondents in the youngest age group (40–49) indicated psychological distress. Some studies have also reported that distress declines with age [27, 29, 39, 40]. The experience of poor mental health may differ between young and old generations, perhaps because of the differences in stressors across the lifespan [29]. In view of the differences in gender roles between Japanese males and females, some specific job-related and family-related stressors should be identified [41, 42]. Moreover, menopause-related symptoms among middle-aged females could be one of the causes of the worst GHQ-12 score levels [43]. Health promotion programmes for middle-aged females would, thus, be required.

Practising two or three kinds of healthy lifestyle behaviours was found to be associated with a reduced risk of psychological distress [23]. As for the results of this and previous studies, healthy lifestyle practices such as getting adequate sleep and exercise could be beneficial for improving mental health conditions [23]. However, it seems that the mechanism connecting SES, lifestyle factors, and mental health is complex and requires longitudinal investigation [27].

This study was based on a large data set, and thus has some important contributions to make. First, the examination of the association of socioeconomic and lifestyle factors with mental health outcomes among working adults was based on a nationally representative sample of over 3000 respondents. Second, this study found significant associations between socioeconomic and lifestyle factors and mental health outcomes after controlling for relevant factors. The results imply the existence of different mechanisms linking socioeconomic and lifestyle factors with mental health conditions among males and females with different SES.

However, the study has several limitations. First, a part of the panel data set was used in a cross-sectional form. Thus, it is not possible to make causal inferences based on these results. It appears essential to discuss causality running in both directions: poor financial conditions may breed psychological distress, and psychological distress may lead to poorer financial conditions [44]. As a next step, analysing the entire panel data set would be beneficial for identifying causal relationships for further discussion. Second, there is the possibility of selection and information bias due to attrition. Respondents who dropped out of the study might have been more likely to be unhealthy or in unfavourable situations. Finally, as the data on SES and lifestyle factors were self-reported, the possibility of social desirability bias and recall bias cannot be excluded.

## Conclusions

The results indicated that socioeconomic and lifestyle factors were associated with mental health conditions. The relationships between mental health conditions and lifestyles significantly differed by household disposable income levels and employment status. Therefore, it is necessary to minimise the health inequalities associated with disposable income levels by developing a social welfare policy and health promotion programmes to prevent poor mental health conditions. This study revealed the differences in healthy lifestyle behaviours between people with low and high SES. Health promotion programmes are needed to foster healthy practices such as adequate sleep, frequent physical exercise, controlled alcohol consumption, and refraining from smoking in different population groups. There might be a complex mechanism of the association between socioeconomic and health-related factors. Further research with respect to the differential associations of gender, age, and SES with health outcomes is required. Determining the complex mechanisms linking mental health conditions and socioeconomic and lifestyle factors would benefit the development of better preventive mental health programmes and a reduction of the risks associated with health and socioeconomic inequalities in different life stages.

## Data Availability

The datasets used and analysed in the current study are available on the website of the Panel Data Research Center at Keio University: https://www.pdrc.keio.ac.jp/en/. The website explains how to register the request form. The centre may approve within a few days on reasonable request.

## Abbreviations

AOR: Adjusted odds ratio
CI: Confidence interval
GHQ-12: 12-item General Health Questionnaire
JHPS/KHPS: Japan Household Panel Survey/Keio Household Panel Survey
OR: Odds ratio
WHO: World Health Organization
